# Reactivation of Herpes Simplex Virus Type 1 (HSV-1) Detected on Bronchoalveolar Lavage Fluid (BALF) Samples in Critically Ill COVID-19 Patients Undergoing Invasive Mechanical Ventilation: Preliminary Results from Two Italian Centers

**DOI:** 10.3390/microorganisms10020362

**Published:** 2022-02-04

**Authors:** Daniele Roberto Giacobbe, Stefano Di Bella, Silvia Dettori, Giorgia Brucci, Verena Zerbato, Riccardo Pol, Ludovica Segat, Pierlanfranco D’Agaro, Erik Roman-Pognuz, Federica Friso, Luigi Principe, Umberto Lucangelo, Lorenzo Ball, Chiara Robba, Denise Battaglini, Andrea De Maria, Iole Brunetti, Nicolò Patroniti, Federica Briano, Bianca Bruzzone, Giulia Guarona, Laura Magnasco, Chiara Dentone, Giancarlo Icardi, Paolo Pelosi, Roberto Luzzati, Matteo Bassetti

**Affiliations:** 1 Department of Health Sciences (DISSAL), University of Genoa, 16132 Genoa, Italy; silvidetto@gmail.com (S.D.); giorgia.brucci@libero.it (G.B.); de-maria@unige.it (A.D.M.); federica.briano91@gmail.com (F.B.); icardi@unige.it (G.I.); matteo.bassetti@unige.it (M.B.); 2 Clinica Malattie Infettive, San Martino Policlinico Hospital-IRCCS for Oncology and Neurosciences, 16132 Genoa, Italy; laura.magnasco@hsanmartino.it (L.M.); chiara.dentone@hsanmartino.it (C.D.); 3 Clinical Department of Medical, Surgical and Health Sciences, University of Trieste, 34127 Trieste, Italy; stefano932@gmail.com (S.D.B.); romanpognuz.erik@gmail.com (E.R.-P.); roberto.luzzati@asugi.sanita.fvg.it (R.L.); 4 Infectious Diseases Unit, Trieste University Hospital, 34127 Trieste, Italy; verena.zerbato@gmail.com (V.Z.); riccardopol91@gmail.com (R.P.); 5 Laboratorio di Riferimento per SARS-CoV-2, Regione Friuli-Venezia Giulia, Azienda Sanitaria Universitaria Integrata Giuliano-Isontina (ASUGI), UCO Igiene e Sanità Pubblica, 34127 Trieste, Italy; ludovica.segat@asugi.sanita.fvg.it (L.S.); pierlanfranco.dagaro@asugi.sanita.fvg.it (P.D.); 6 Department of Perioperative Medicine, Intensive Care and Emergency, Cattinara Hospital, Trieste University, 34149 Trieste, Italy; federica.friso.5@gmail.com (F.F.); umberto.lucangelo@asuits.sanita.fvg.it (U.L.); 7 Clinical Pathology and Microbiology Unit, “San Giovanni di Dio” Hospital, 88900 Crotone, Italy; luigi.principe@gmail.com; 8 Department of Surgical Sciences and Integrated Diagnostics (DISC), University of Genoa, 16132 Genoa, Italy; lorenzo.ball@unige.it (L.B.); chiara.robba@unige.it (C.R.); npatroniti@gmail.com (N.P.); ppelosi@hotmail.com (P.P.); 9 Anaesthesia and Intensive Care, San Martino Policlinico Hospital-IRCCS for Oncology and Neurosciences, 16132 Genoa, Italy; denise.battaglini@hsanmartino.it (D.B.); brunettimed@gmail.com (I.B.); 10 Department of Medicine, University of Barcelona, 08007 Barcelona, Spain; 11 Hygiene Unit, San Martino Policlinico Hospital-IRCCS for Oncology and Neurosciences, 16132 Genoa, Italy; bianca.bruzzone@hsanmartino.it (B.B.); giuly.guarons@hotmail.it (G.G.)

**Keywords:** HSV-1, herpes simplex virus, reactivation, intensive care, COVID-19, SARS-CoV-2, BALF

## Abstract

Reactivation of herpes simplex virus type 1 (HSV-1) has been described in critically ill patients with coronavirus disease 2019 (COVID-19) pneumonia. In the present two-center retrospective experience, we primarily aimed to assess the cumulative risk of HSV-1 reactivation detected on bronchoalveolar fluid (BALF) samples in invasively ventilated COVID-19 patients with worsening respiratory function. The secondary objectives were the identification of predictors for HSV-1 reactivation and the assessment of its possible prognostic impact. Overall, 41 patients met the study inclusion criteria, and 12/41 patients developed HSV-1 reactivation (29%). No independent predictors of HSV-1 reactivation were identified in the present study. No association was found between HSV-1 reactivation and mortality. Eleven out of 12 patients with HSV-1 reactivation received antiviral therapy with intravenous acyclovir. In conclusion, HSV-1 reactivation is frequently detected in intubated patients with COVID-19. An antiviral treatment in COVID-19 patients with HSV-1 reactivation and worsening respiratory function might be considered.

## 1. Introduction

Critically ill patients with coronavirus disease 2019 (COVID-19) pneumonia requiring invasive mechanical ventilation are at risk of developing bacterial, fungal, and viral superinfections [1,2,3,4]. Reactivation of herpes simplex virus type 1 (HSV-1) has been described in intubated critically ill patients with coronavirus disease 2019 (COVID-19) pneumonia [2,3,5,6,7,8]. 

Both predictors and prognostic implications of HSV-1 reactivation in this peculiar population remain largely elusive, making its management highly heterogenous in terms of prophylactic and therapeutic approaches [2,3,5]. In the present two-center retrospective experience, we aimed to assess the cumulative risk of HSV-1 reactivation detected on bronchoalveolar fluid (BALF) samples in invasively ventilated COVID-19 patients with worsening respiratory function, as well as to exploratorily assess the predictors of HSV-1 reactivation and its possible prognostic impact.

## 2. Materials and Methods

The present retrospective study was conducted in two centers in Italy: (i) IRCCS Ospedale Policlinico San Martino in Genoa; (ii) ASUGI University Hospital in Trieste. The study period was from 1 April 2020 to 31 May 2021. The primary objective was to assess the cumulative risk of HSV-1 reactivation detected on deep respiratory samples in critically ill patients with COVID-19 and worsening respiratory function (according to treating physicians’ judgment that prompted the search for HSV-1 reactivation). The secondary objectives were to assess the predictors of HSV-1 reactivation detected on BALF samples and its possible prognostic impact.

In line with the study primary objective, the study population included adult, critically ill patients with COVID-19 undergoing invasive mechanical ventilation who fulfilled all the following inclusion criteria: (i) they underwent bronchoscopy at the time of, or soon after, orotracheal intubation with quantitative molecular testing for HSV-1 on bronchoalveolar lavage fluid (BALF); (ii) their first molecular testing on BALF was negative (no HSV-1-DNA copies detected); (iii) they underwent at least another quantitative molecular testing for HSV-1 on a BALF sample within 30 days after the first bronchoscopy because of worsening respiratory function.

SARS-CoV-2 infection was defined in presence of at least one positive real-time polymerase chain reaction on respiratory specimens. HSV-1 reactivation was defined as detection of HSV-1-DNA ≥ 10^4^ copies/mL on BALF samples [9].

### Statistical Analysis

The cumulative risk of HSV-1 reactivation in the study population was calculated by means of the Aalen–Johansen method. HSV-1 reactivation was considered as the event of interest, death as a competing event, and discharge from the intensive care unit as a right-censoring event. Time of origin was the day of the initial negative molecular test for HSV-1-DNA on BALF samples.

A first secondary analysis was the identification of predictors of HSV-1 reactivation. To this aim, demographic and clinical variables were tested for their association with HSV-1 reactivation by univariable Cox regression models, to estimate the related unadjusted cause-specific hazard ratio (HR). Time of origin was the day of the initial negative molecular test for HSV-1-DNA on BALF, and the maximum follow-up was 30 days after the time of origin. Factors potentially associated with HSV-1 reactivation in univariable comparisons (*p* < 0.10) were included in a multivariable Cox regression model, for estimating their adjusted cause-specific HR.

Another secondary analysis was aimed to exploratorily assess the possible impact of HSV-1 reactivation on mortality. To this aim, HSV-1 reactivation was modeled as a time-varying variable and tested for its possible association with mortality in a univariable Cox regression model with time of origin as the day of the initial BALF testing negative for HSV-1-DNA, and with 30 days as the maximum follow up. Then, independent of the results of univariable analysis, HSV-1 reactivation as a time-varying variable was included in a multivariable Cox regression model together with the following other time-varying covariates that could also justify worsening of lung function due to superinfection: (i) cytomegalovirus (CMV) reactivation (defined as detection of CMV-DNA ≥ 10^4^ copies/mL in BALF samples in patients with worsening respiratory function); (ii) at least possible COVID-19-associated pulmonary aspergillosis (defined according to the 2020 ECMM/ISHAM consensus criteria [10]); (iii) ventilator-associated bacterial pneumonia (VABP), as previously defined [11]. *Pneumocystis jirovecii* pneumonia (PJP) was not included in the model because no patients in the study population developed PJP according to molecular BALF testing.

## 3. Results

**Figure 1:**
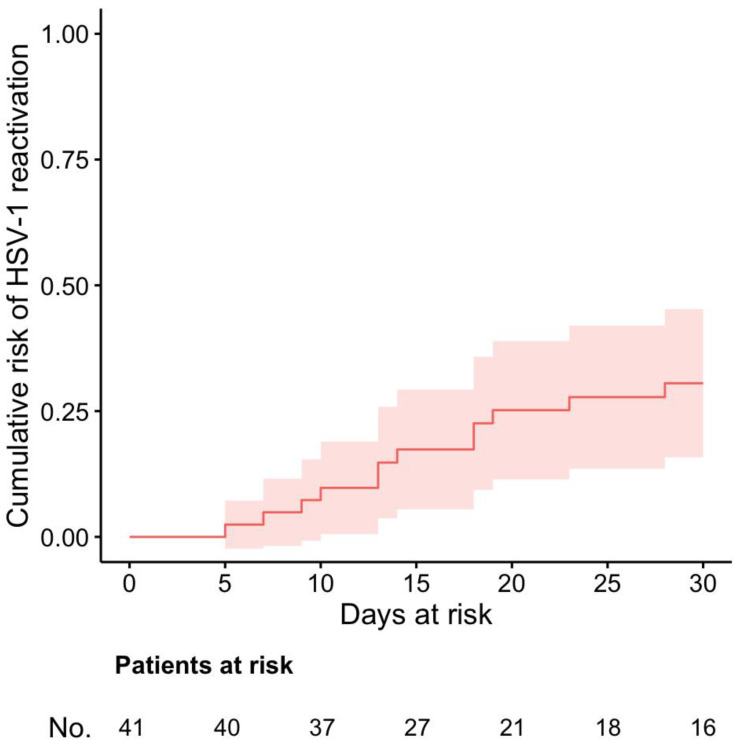
Cumulative risk of HSV-1 reactivation in the study population, estimated by means of the Aalen–Johansen method, with HSV-1 reactivation as the event of interest, discharge from the intensive care unit as a right-censoring event, and death as a competing event. The time of origin was the day of the initial negative molecular test for HSV-1-DNA on BALF samples.

Overall, 41 patients met the study inclusion criteria (see Supplementary Materials Figure S1). Their demographic and clinical characteristics are shown in Table 1. Overall, 12/41 patients developed HSV-1 reactivation (29%). This crude percentage is in line with the cumulative risk of developing HSV-1 reactivation reached at 30 days and measured with cumulative incidence curve (Figure 1).

No independent predictors of HSV-1 reactivation were found at univariable and multivariable analyses (Table 2). Concomitant search for HSV-1-DNA in blood was performed in 13/41 patients (32%). Of these 13 patients tested for HSV-1-DNA on blood, 12 had HSV-1 reactivation on BALF, while 1 had no HSV-1 reactivation on BALF. Blood HSV-1-DNA was positive in 3/13 patients. All the three patients with positive HSV-1-DNA on blood had HSV-1 reactivation detected on BALF samples.

The crude 30-day mortality in the study population was 32% (13/41), and 25% in patients with HSV-1 reactivation (3/12). No association was found between HSV-1 reactivation and mortality, both in univariable and in multivariable Cox regression models (Table 3). Overall, one patient with HSV-1 reactivation did not receive anti-HSV-1 antiviral therapy, whereas 11/12 were treated with intravenous acyclovir (92%).

## 4. Discussion

Up to 29% of critically ill patients with acute respiratory failure due to COVID-19 and no detection of HSV-1-DNA on BALF samples at the time of intubation may develop HSV-1 reactivation within the subsequent 30 days. Further, no independent predictors of HSV-1 reactivation were identified, nor was association between HSV-1 reactivation and mortality. Most of the patients with HSV-1 reactivation received antiviral therapy with intravenous acyclovir.

In line with our results, two recent single-center studies reported that in hospitalized patients with severe or critical COVID-19 pneumonia, the frequency of HSV-1 reactivation was 31% on respiratory samples [12], and 30% in the plasma [2]. Differently from our study, an independent association was found between steroid treatment and HSV-1 reactivation [2]. These apparently opposite findings may be explained by the fact that almost all patients in our cohort (39/41) received steroid treatment, thereby drastically reducing our ability to assess their role as a predisposing factor for HSV-1 reactivation in our study. However, from a practical perspective, it is worth noting that steroid treatment is nowadays employed in almost all patients with severe or critical COVID-19 pneumonia. Therefore, there may be more interest in identifying other modifiable predictors of HSV-1 reactivation that could impact possible preventive strategies, for example, nonsteroid anti-inflammatory treatments, such as tocilizumab. In this regard, while we administer tocilizumab in selected critically ill patients with COVID-19 pneumonia [13,14], none of the patients who met the inclusion criteria for the present study were treated with tocilizumab, and, also, no clear association was found in the previously mentioned study by Franceschini and colleagues [2]. With regards to other possible predictors besides steroid and nonsteroid anti-inflammatory agents, while recognizing the low power of our preliminary analysis (that could justify the lack of statistical significance), it is also worth noting that no large effect sizes were registered for potential predictors of HSV-1 reactivation in our study population, thereby suggesting that there may be no singular characteristic or therapeutic approach majorly influencing the risk as a standalone predictor.

Our secondary objective was to assess the impact of HSV-1 reactivation on mortality of critically ill COVID-19 patients undergoing invasive mechanical ventilation. We designed the present study with a clinician-oriented approach, i.e., by following our real-life practice in which HSV-1 reactivation was searched for in the presence of worsening of respiratory function. This increased the chance of detecting a clinically significant reactivation with organ damage, compared with any possible assessment of reactivation by routine screening of HSV-1-DNA (independent of clinical conditions), and thus to better assess any potential impact of HSV-1 reactivation on mortality. When more properly modeling HSV-1 reactivation as a time-varying factor, we did not detect an association with mortality. This result should be interpreted considering that almost all patients with HSV-1 reactivation in our cohort (12/13) received intravenous acyclovir treatment. Our findings may support the hypothesis that, if existent, any unfavorable prognostic impact of HSV-1 reactivation in intubated COVID-19 patients could be mitigated (or cancelled) by proper antiviral treatment. Another possibility is that the association with mortality of HSV-1 reactivation in critically ill COVID-19 patients may be driven by blood reactivation and not by respiratory reactivation [12]. In our study, we detected blood HSV-1-DNA only in three patients with reactivation detected on BALF. A possible speculative hypothesis to be further explored may be the lung inhalation after oropharyngeal reactivation, without significant pulmonary damage [15]. Regarding mortality, it should be considered that the available literature on the prognostic impact of HSV-1 reactivation in non-COVID-19 critically ill populations (which also remains controversial) may be not extrapolated directly to COVID-19 patients, owing to the possibly different immunological background [6,15,16,17,18,19,20].

The present study has several limitations. Besides the retrospective design, the other, most important limitation is the small sample size, thus our results should be necessarily considered as preliminary and hypothesis-generating. Second, the study was not designed to also investigate the possible prognostic impact of HSV-1 reactivation without concomitant worsening of respiratory function, which may be relevant for more comprehensively discussing any possible role of prophylaxis, or of potential pre-emptive approaches. Third, we had limited data about concomitant HSV-1-DNA counts in blood (mostly searched in patients with positive HSV-1-DNA on BALF), that precluded an assessment of their potential impact on mortality in our cohort. Fourth, it should be necessarily noted that no defined criteria currently exist for diagnosing HSV pneumonia in the absence of histopathological confirmation, which was unavailable in our study [21]. From this standpoint, we must recognize that our choice of the quantitative cut-off for HSV-1 reactivation was arbitrary, although motivated by the concomitant presence of a consistent clinical picture, and also that higher other cut-offs have been previously proposed in non-COVID-19 patients [9,22,23]. Finally, we should acknowledge that we cannot completely exclude novel infection/cross-transmission of HSV-1 instead of reactivation, as we have serological HSV-1 status at admission only for 12 patients (all with positive IgG). However, since all of them were positive for previous infection, and HSV-1 infection is frequently acquired in childhood (with 50% to 85% of adults being seropositive [24,25,26]), this may support our definition of reactivation, which is also in line with other experiences in COVID-19 patients [2,3].

In conclusion, HSV-1 reactivation is frequently detected in critically ill patients with COVID-19 undergoing invasive mechanical ventilation. An antiviral treatment in COVID-19 patients with HSV-1 reactivation and worsening respiratory function might be considered.

## Figures and Tables

**Table 1:** Characteristics of the study population.

Variable	Total 41 (100)
**Patient variables**	
Age in years, median (IQR)	65 (60–70)
Male sex	33 (80)
Body mass index, median (IQR)	27 (25–31)
Diabetes mellitus	8 (20)
Hypertension	23 (56)
Chronic obstructive pulmonary disease	5 (12)
End-stage renal disease ^a^	4 (10)
Moderate/severe liver failure ^b^	0 (0)
Solid cancer	2 (5)
Hematological malignancy	1 (2)
HIV infection	0 (0)
Charlson comorbidity index, median (IQR)	3 (2–4)
**Characteristics at time of intubation**	
Blood CD4+ T cells (cell/mm^3^), median (IQR) ^c^	194 (125–260)
Blood CD8+ T cells (cell/mm^3^), median (IQR) ^c^	57 (51–94)
Blood CD4+/CD8+ T cells ratio, median (IQR) ^c^	2.6 (1.5–3.5)
Serum D-dimer (ng/mL), median (IQR) ^d^	2349 (1262–5477)
Serum albumin (g/L), median (IQR) ^d^	22.9 (19.6–27.9)
SOFA score, median (IQR)	5 (4–6)
Days of NIV/CPAP before intubation, median (IQR)	4 (2–6)
**COVID-19 therapy**	
Steroid treatment ^e^	38 (93)
Tocilizumab	0 (0)
Remdesivir	21 (51)

Results are reported as number of patients (%) unless otherwise indicated. COVID-19, coronavirus disease 2019; CPAP, continuous positive airway pressure; NIV, noninvasive ventilation; HIV, human immunodeficiency virus; IQR, interquartile range; SOFA, sequential organ failure assessment. ^a^ Defined as estimated glomerular filtration rate < 15 mL/min/1.73 m^2^; ^b^ Defined as compensated or decompensated liver cirrhosis; ^c^ Information available for 17/41 patients; ^d^ Information available for 39/41 patients; ^e^ Dexamethasone 6–8 mg/die, methylprednisolone 0.5–1 mg/kg/die, or equivalent dosages of other steroids.

**Table 2:** Univariable and multivariable analyses of potential predictors of HSV-1 reactivation *.

Variable	Unadjusted Cause-Specific HR (95% CI)	*p*	Adjusted Cause-Specific HR (95% CI) ***	*p*
Age in years	1.00 (0.96–1.05)	0.860		
Male sex	1.60 (0.35–7.30)	0.547		
Body mass index	0.99 (0.90–1.10)	0.860		
Diabetes mellitus	0.31 (0.04–2.47)	0.274		
Hypertension	1.53 (0.48–4.84)	0.473		
Chronic obstructive pulmonary disease	0.04 (0.00–42.50)	0.039	0.99 (0.10–4.41)	0.991
End-stage renal disease	0.71 (0.09–5.53)	0.746		
Solid cancer	4.28 (0.92–19.90)	0.063	1.38 (0.01–14.85)	0.839
Hematological malignancy **	1.05 (0.01–8.05)	0.973		
Charlson comorbidity index	1.06 (0.77–1.45)	0.730		
Blood CD4+ T cells (cell/mm^3^)	1.00 (1.00–1.01)	0.531		
Blood CD8+ T cells (cell/mm^3^)	1.01 (1.00–1.01)	0.290		
Blood CD4+/CD8+ T cells ratio	0.91 (0.68–1.22)	0.540		
Serum D-dimer (ng/mL)	1.00 (1.00–1.00)	0.905		
Serum albumin (g/L)	0.95 (0.85–1.07)	0.403		
SOFA score	0.64 (0.40–1.03)	0.067	0.84 (0.55–1.25)	0.408
Days of NIV/CPAP before intubation	1.04 (0.91–1.20)	0.572		
Steroid treatment **	0.31 (0.09–1.60)	0.110		
Remdesivir	0.88 (0.28–2.73)	0.823		

COVID-19, coronavirus disease 2019; CI, confidence intervals; CPAP, continuous positive airway pressure; NIV, noninvasive ventilation; HR, hazard ratio; SOFA, sequential organ failure assessment. * The variables moderate/severe liver failure, human immunodeficiency virus (HIV) infection, and tocilizumab therapy were not included in the model because they were not present in patients fulfilling inclusion criteria for this specific study. ** Standard univariable Cox regression models did not converge in the presence of very small groups *(n =* 1 for patients with hematological malignancies and *n* = 2 for patients not receiving steroid treatment). The presented unadjusted HR and the 95% CI for these variables were obtained by means of penalized, univariable Cox regression models with Firth’s correction, built using the coxphf package for R Statistical Software (R Foundation for Statistical Computing, Vienna, Austria). *** Nonconvergence was observed with the standard multivariable Cox regression model. Therefore, the presented adjusted HRs and their 95% CI were obtained by means of penalized, multivariable Cox regression models with Firth’s correction, built using the coxphf package for R Statistical Software (R Foundation for Statistical Computing, Vienna, Austria).

**Table 3:** Univariable and multivariable analyses of the potential impact on mortality of pulmonary infectious events in intubated COVID-19 patients.

Variables *	Unadjusted HR (95% CI)	*p*	Adjusted HR (95% CI)	*p*
HSV-1 reactivation	1.43 (0.38–5.46)	0.598	1.07 (0.24–4.86)	0.928
CMV reactivation	0.43 (0.09–1.99)	0.280	0.25 (0.05–1.27)	0.096
CAPA	1.31 (0.29–5.98)	0.726	0.68 (0.11–4.16)	0.679
VABP	2.34 (0.65–8.51)	0.195	3.73 (0.93–14.88)	0.063

CAPA, coronavirus disease 2019-associated pulmonary aspergillosis; COVID-19, coronavirus disease 2019; CI, confidence intervals; CMV, cytomegalovirus; HSV-1, herpes simplex virus 1; HR, hazard ratio; VABP, ventilator-associated bacterial pneumonia. * All variables were included in Cox regression models as time-varying factors (see study methods).

## Data Availability

The data presented in this study are available on reasonable request from the corresponding author.

## Supplementary Materials

The following supporting information can be downloaded at: www.mdpi.com/article/10.3390/microorganisms10020362/s1, Figure S1: Flow-chart of the patient inclusion process.
