# Supplementary figures and images for: Reactivation of Herpes Simplex Virus Type 1 (HSV-1) Detected on Bronchoalveolar Lavage Fluid (BALF) Samples in Critically Ill COVID-19 Patients Undergoing Invasive Mechanical Ventilation: Preliminary Results from Two Italian Centers
Source: Microorganisms. 2022 Feb 4;10(2):362. doi: 10.3390/microorganisms10020362 (PMC8875622; doi:10.3390/microorganisms10020362)

### Supplementary figure S1. Flow-chart of the patient inclusion process

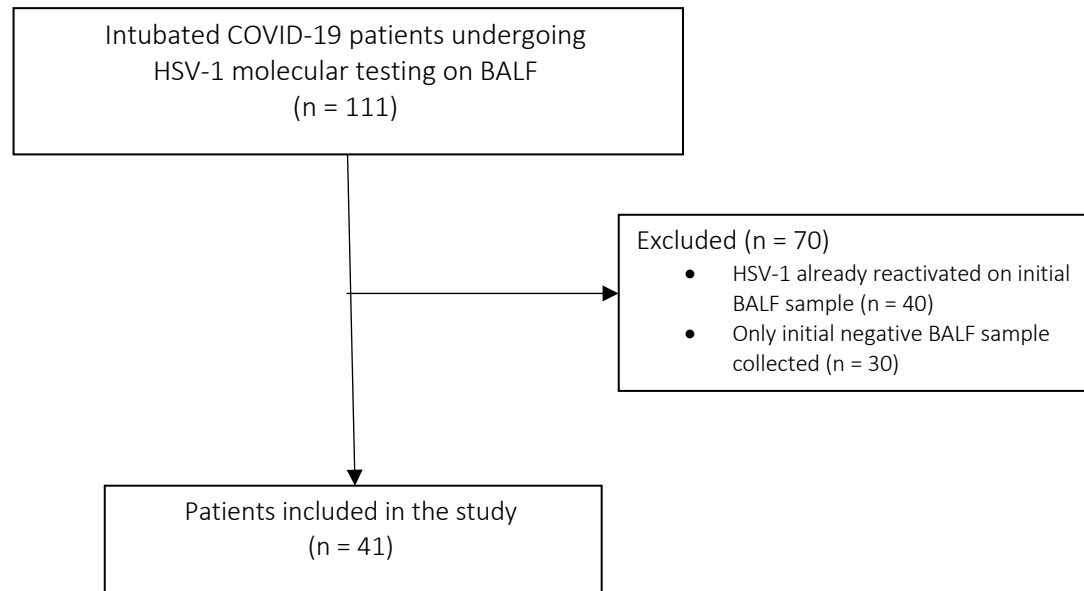

Supplement: Supplementary file 1 [file microorganisms-10-00362-s001.zip › supplementary figure S1.pdf]
